# Feasibility, Acceptability, and Preliminary Effectiveness of a Combined Digital Platform and Community Health Worker Intervention for Patients With Heart Failure: Protocol for a Randomized Controlled Trial

**DOI:** 10.2196/55687

**Published:** 2024-02-06

**Authors:** Jocelyn Carter, Natalia Swack, Eric Isselbacher, Karen Donelan, Anne Thorndike

**Affiliations:** 1 Division of General Internal Medicine Massachusetts General Hospital Boston, MA United States; 2 Department of Medicine Massachusetts General Hospital Boston, MA United States; 3 Corrigan Minehan Heart Center Massachusetts General Hospital Boston, MA United States; 4 Heller School for Policy and Management Brandeis University Waltham, MA United States

**Keywords:** heart failure, digital platform, remote monitoring, home-based care, health worker, social needs care, community health worker

## Abstract

**Background:**

Interventions focused on remote monitoring and social needs care have shown promise in improving clinical outcomes for patients with heart failure (HF). However, patient willingness to use technology as well as concerns about access in underresourced settings have limited digital platform implementation and adoption. There is little research in HF populations examining the effect of a combined digital and social needs care intervention that could enhance patient engagement in digital platform use while closing gaps in care related to social determinants of health. Here, we describe the protocol for a clinical trial of a digitally enabled community health worker intervention designed for patients with HF.

**Objective:**

This study aims to describe the protocol for a randomized controlled trial assessing the acceptability, feasibility, and preliminary effectiveness of an intervention that combines remote monitoring with a digital platform and community health worker (CHW) social needs care for patients with HF who are transitioning from hospital to home. Given the elevated morbidity and mortality, identifying comprehensive and patient-centered interventions at the time of hospital care transitions that can improve clinical outcomes, impact cost, and augment the quality of care for this cohort is a priority.

**Methods:**

This trial randomized adult inpatient participants (n=50) with a diagnosis of HF receiving care at a single academic health care institution to the 30-day intervention (digital platform+CHW pairing+usual care) or the 30-day control (CHW pairing+usual care) arms. All study participants completed baseline questionnaires and 30-day exit interviews and questionnaires. The primary outcomes will be acceptability, feasibility, and preliminary effectiveness.

**Results:**

This clinical trial opened for enrollment in September 2022 and was completed in June 2023. Initial results are expected to be published in the spring of 2024, and analysis is currently underway. Feasibility outcome measures will include the use rates of the biometric sensor (average hours per day), the digital blood pressure monitor (average times per day), the weight scale (average times per day), and the completion of the symptoms questionnaire (average times per day). The acceptability outcome will be measured by the patients’ response to the truthfulness of the statement that they would be willing to use the digital platform in the future (response options: very true, somewhat true, or not true). Preliminary effectiveness will be measured by tracking 30-day clinical outcomes (hospital readmissions, emergency room visits, and missed primary care and cardiology appointments).

**Conclusions:**

The results of this investigation are expected to contribute to our understanding of the use of digital interventions and the implementation of supportive home-based social needs care to enhance engagement and the potential effectiveness of clinically focused digital platforms. These results may inform the construction of a future multi-institutional trial designed to test the true effectiveness of this intervention in HF.

**Trial Registration:**

ClinicalTrials.gov NCT05130008; https://clinicaltrials.gov/study/NCT05130008

**International Registered Report Identifier (IRRID):**

DERR1-10.2196/55687

## Introduction

### Background

Heart failure (HF) is one of the most common causes of 30-day readmissions and is a morbid and burdensome disease for patients [1]. Factors related to clinical complexity [2,3] and unmet social needs [4] have been identified as key culprits contributing to rising costs of HF care associated with HF exacerbations and recurrent hospitalizations [5]. While individual interventions focused on digital solutions to augment either clinical care [6-8] or home-based care [9-11] to address unmet social needs have the potential to improve HF clinical outcomes, these interventions are challenged by specific barriers that limit their effectiveness.

Digital platforms with remote monitoring capabilities can track fundamental biometrics, such as heart rate, blood pressure, and body weight, which are critical for the home management of HF [12-15]. These biometrics can be used to evaluate clinical status at home with automated reporting back to patient teams [16-19]. Some platforms also offer daily symptom questionnaires and even embed artificial intelligence algorithms with the ability to tailor alerts to individual patient norms, which can improve platform accuracy and precision [13]. These digital platform capabilities have improved the detection of markers signaling clinical decline (eg, changes in weight, activity tolerance, heart rates, blood pressure, and symptomatology), and a number of studies have demonstrated an early benefit of digital platform use for clinically complex patients, including those managing HF at home [6,8,20,21].

However, the integration of digital platforms among patients with HF has been incremental, at best, for reasons ranging from infrastructure limitations of health care institutions to patient-related barriers [22,23]. Specifically, several patient care barriers have been identified including knowledge gaps, lack of willingness to gain familiarity with technology, reduced health care access, and marginal internet connectivity [22,23]. Concordantly, valid concerns about the exclusion of digital platform use in low-resourced, aging, or less technology-facile populations exist [19]. A number of these challenges could be resolved by integrating a home-based human resource, with basic knowledge of the digital platform who could also deliver social needs care.

Home-based care that focuses on social needs is often delivered by community health workers (CHWs) [24,25]. CHWs, with basic knowledge of chronic conditions, can address unmet social needs and reinforce clinical care plans in ways that improve outcomes by bolstering connections to clinical care teams [26]. Specifically, CHW outreach includes telephone calls; home visits; health care coaching; accompaniment to clinic visits; and identification of low or no-cost resources to close gaps in care related to food insecurity, transportation, rental assistance, or other unmet social needs. CHWs can also provide elbow-to-elbow support with the completion of insurance forms or agency applications. Through motivational interviewing, goal setting, and psychosocial support, CHWs can work closely with patients to identify and address logistical barriers to care [27]. While the evidence base for CHW-focused interventions to improve outcomes and reduce readmissions for patients with chronic diseases including HF is robust [10,11,28-31], CHWs generally rely on one-to-one in-person or phone or text-based patient interactions without symptom or biometric monitoring [32]. In this way, CHW care remains largely siloed without the tools needed to care for larger, clinically complex populations. As such, CHWs inadvertently spend more time with patients who are healthier and engaged in care and less time with patients who are harder to reach and would benefit from early intervention [33,34]. While manageable in smaller cohorts and studies, this can stifle the impact of CHWs on clinical outcomes when scaled.

A combined intervention with a digital platform and CHW care (ie, digitally enabled CHW care) could address important barriers associated with platform use. Specifically, CHW care, through home visits, phone calls, and connections to care teams, can address digital platform knowledge gaps and connectivity issues [27]. CHWs can also act as navigators for digital platforms by encouraging use and engagement that can improve platform usability and adoption. In this way, CHWs are uniquely positioned to enhance the use and reach of digital platforms. In addition, pairing patients at high risk for readmission, especially those with HF, with a CHW and a digital platform could address key barriers to CHW care delivery related to the historical reliance on one-to-one outreach [35]. Digital platforms can better inform the timing and prioritization of CHW outreach with real-time access to streamlined basic biometric data (weights, steps taken per day, or a daily clinical score) and patient response data (collected in a daily questionnaire). This access can augment the ability of CHWs to connect patients to clinical care teams earlier for interventions that can potentially prevent hospital readmissions or emergency department (ED) visits.

This trial, based on a single-arm observational study performed prior [27], will study the effect of a digitally enabled CHW intervention created for patients with HF. In this way, the study addresses a gap in current knowledge of home-based care for patients with HF as one of the first randomized controlled trials (RCTs) examining the effect of a digital platform combined with social needs care delivery from a CHW in patients with HF. This work is important because it seeks to reduce hospital readmissions for HF, which is imperative for Centers for Medicare and Medicaid stakeholders [36]. This study intervention also occurs at the time of hospital discharge, a particularly vulnerable time point as patients are transitioning out of the hospital. The results of this clinical trial may also contribute to a novel and innovative model for home-based care in a high-risk HF population. Specifically, this study will add value to ongoing conversations about the applications and implementation of digital solutions and unmet social needs, particularly in chronic disease populations with serious illness.

The goal of this trial is to determine if a remote monitoring and social needs care intervention deployed at the time of hospital discharge is feasible, acceptable, and can demonstrate preliminary effectiveness relevant to clinical outcomes (hospital readmissions, ED visits, and missed appointments). We expect to see a higher degree of engagement and adherence to clinical care among those assigned to digitally enabled CHW care compared to those assigned to CHW care alone. The methodology described here will add insight into the implementation of similar digital interventions for patients transitioning from hospital to home with HF.

### Primary Objective

The objective of this trial is to assess the acceptability, feasibility, and preliminary effectiveness of a digitally enabled CHW intervention.

### Hypothesis

The central hypothesis is that pairing patients with a digitally enabled CHW intervention that addresses clinical, social, and behavioral barriers to HF care will (1) be feasible for patients with HF at home, (2) be acceptable for patients with HF at home, and (3) demonstrate preliminary effectiveness in improving clinical outcomes.

## Methods

### Study Overview and Design

**Figure 1:**
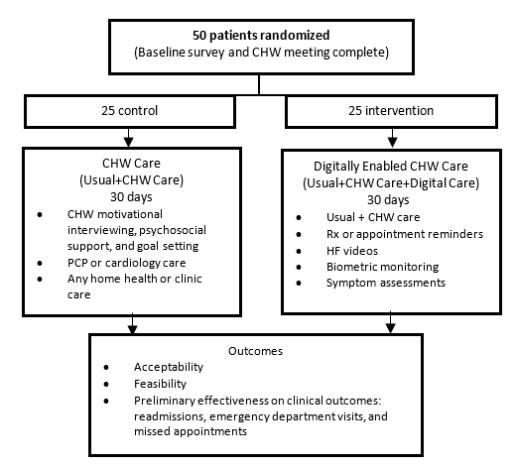
Trial design, study arm assignment, and outcomes of a pilot randomized controlled trial. CHW: community health worker. PCP: primary care provider. Rx: medical prescription. HF: heart failure.

An RCT design was applied to evaluate the intervention (digital platform+CHW+usual care) group compared to the control (CHW+usual care) group during the 30 days after discharge from the hospital to home. Figure 1 describes the study’s procedural flow. Eligible patients were screened via the electronic medical record (EMR) on 8 inpatient study floors (6 internal medicine floors and 2 cardiology floors) in a single health care institution. Research staff approached patients after obtaining permission from bedside nursing. After verifying eligibility and introducing the study design, interested patients completed consent processes and all enrollment questionnaires. Participants were randomized to the intervention or the control arm for the 30-day intervention and study period. Both intervention and control participants were contacted by their assigned CHW within 24 weekday hours of enrollment and received teaching via an American Heart Association–sponsored HF patient education tool (educational control). Intervention participants received study equipment and were oriented to the use of all platform components by research staff prior to hospital discharge. All enrolled participants completed an exit questionnaire and interview via phone at the end of the 30-day intervention.

### Clinical Setting of Patient Population

Massachusetts General Hospital (MGH) is a 999-bed academic medical center in Massachusetts with 75,000 to 100,000 hospital admissions each year. The MGH Corrigan Minehan Heart Center serves over 27,000 patients with cardiac disease each year, many of whom have HF. As an accountable care organization and medical home in partnership with the Mass General Brigham system, MGH inpatient teams are comprised of full and part-time staff clinicians, including board-certified internists or cardiologists, residents, fellows, nurses, advanced clinicians (nurse practitioners and physician assistants), and students. The majority of inpatients are supported by Medicare or Medicaid-based insurance products.

### Subject Eligibility and Recruitment Strategy

Eligibility criteria were established based on prior clinical trials and qualitative studies focused on care transitions from hospital to home [27,37-39]. Eligibility criteria for participants who were approached and introduced to the study while hospitalized included the following: being 18 years and older, living within a 50-mile radius of MGH, having a diagnosis of HF listed in the EMR problem list, a history of ≥1 hospitalization within the previous 12 months, a clinician managing their HF, cognitive ability to participate in the intervention, and English fluency. Patients were ineligible if they had an active alcohol or substance use disorder, were living in a long-term care facility, were unable to provide consent, had invoked health care proxy, or had prisoner status. Research staff attempted to enroll patients up to 3 times if they were unsure or unable to be engaged in the initial approach.

### CHW Training and Supervision

Extensive experience training CHWs gained during our prior CHW clinical trial was applied for CHW training purposes [9]. CHW staff participating in the trial were trained in the core competencies of CHW care delivery for HF and other common diagnoses associated with hospital readmissions (eg, pneumonia, atrial fibrillation, and pulmonary disease). These CHW core competencies included motivational interviewing, behavioral change, and psychosocial support. Additional skill sets and activity building occurred for medication reconciliation, common management and postdischarge follow-up plans, general American Heart Association guideline–concordant care, patient educational tools, and commonly used community resources. CHW supervision was led by a CHW manager experienced in supervising CHWs caring for clinically, socially, and behaviorally complex patients. Supervision occurred through daily huddles (with the CHW staff supervisors) and weekly meetings with the CHW staff supervisors and the principal investigator (JC). All clinical aspects of CHW care were also supervised by the principal investigator.

**Figure 2:**
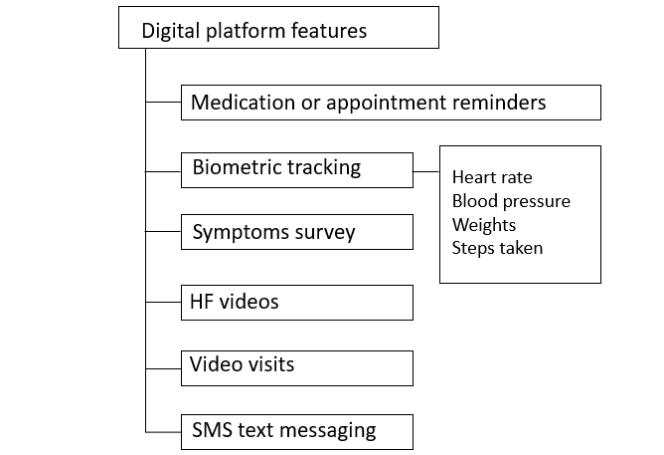
Procedural workflow overview for study participants. CHW: community health worker.

**Figure 3:**
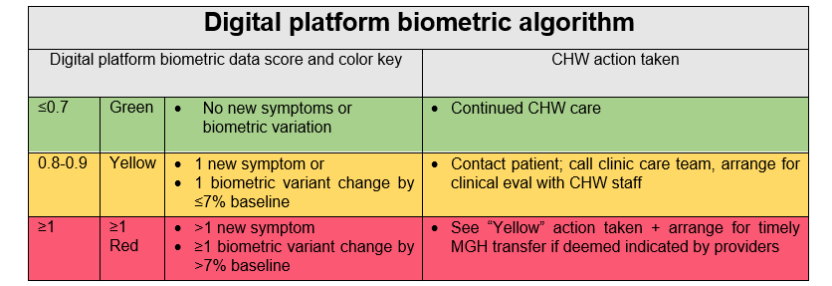
Digital platform components (intervention arm only). HF: heart failure.

For the intervention arm, CHW staff were also trained on the use of the digital platform. The training was fulfilled using participatory methods, case scenarios, and video clips for optimal teaching and application for the patient-facing mobile app as well as the team dashboard. Specific training on the digital platform features (Figure 2) was designed to enhance communication and monitoring in concert with the application of core competencies and skill sets associated with traditional CHW care. This occurred during multicomponent didactics involving feedback, consultation, and supervision. Extensive role play and case studies with simulation experiences for troubleshooting and technology-based challenges were performed over a 14-day period. After training, simulation and behavioral interviewing–based proficiency testing were used to evaluate both CHW best practices and digital platform proficiency. CHW staff were also trained on how to interpret digital platform symptom assessments and biometric monitoring. In the platform, these symptoms and biometrics were translated into a color-coded schematic (Figure 3: green=no new symptoms or biometric variance or clinical symptom score of ≤0.7; yellow=1 new symptom or clinical score of 0.8-0.9; red= more than 1 new symptom or a clinical score of ≥1.0). For any participants with a yellow categorization on any given day, CHWs (1) contacted the participant to verify symptoms and ask probing questions, (2) contacted the participant’s designated care clinic, and (3) coordinated a patient visit with CHW staff or a visiting home nurse for expedited clinical evaluation. For participants with a red categorization, all yellow categorization steps were followed and CHWs partnered with the clinical care team to arrange for expedited transfer to MGH or in-home evaluation if clinically indicated per the clinical care team providers.

### Control

**Figure 4:**
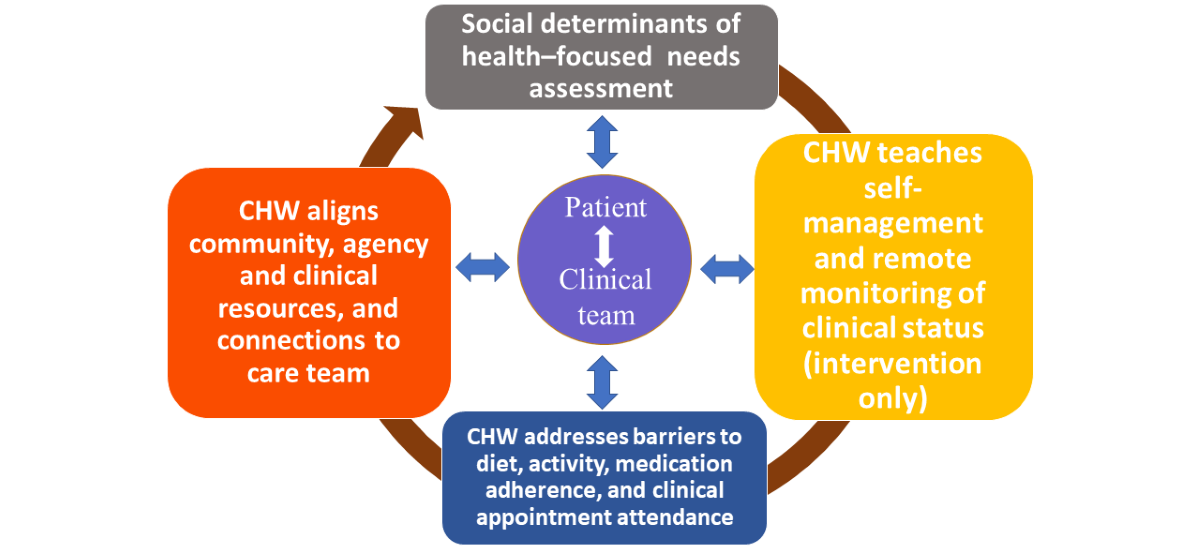
Color schematic depicting the relationship between the biometric clinical status score generated by the digital platform algorithm and the action taken by CHW staff. CHW: community health worker; MGH: Massachusetts General Hospital.

Participants were contacted routinely by CHW staff to review medication adherence, nutrition, physical activity, symptoms, and clinic appointments and discuss any unmet social needs. CHW staff (n=1) paired with enrolled participants reviewed individual participant’s discharge care plans and integrated the patient’s clinical, social, and behavioral goals with clinical care plans. As such, CHWs also identified resources to reduce gaps in care caused by unmet social needs and connected patients to clinical care teams for clinical questions. One CHW with expertise in CHW core competencies [25] (motivational interviewing, goal setting, behavior change, and psychosocial support) delivered the control arm treatment. Daily huddles occurred to discuss patient interactions and plans for goal achievement. CHW staff documented all participant encounters in the EMR. In addition, all CHW interactions were logged in a web-based research team REDCap (Research Electronic Data Capture; Vanderbilt University) database. All social, behavioral, and clinical activities (clinical care team and community agency interactions, as well as time spent engaged in phone, in-person, and email modalities) were tracked. Pre-existing clinical team members were copied on all EMR notes and contacted directly, when necessary, by the CHW or supervisory staff during the intervention. Figure 4 shows an overview of the study participant workflow.

### Intervention

Enrolled intervention participants were introduced to the digital platform features prior to hospital discharge: an HF mobile phone app within a smartphone (Android) that included a daily checklist for patients, educational HF videos, a portal CHW video visits, and a daily symptom questionnaire. In addition, participants were given a digital blood pressure monitor, a digital weight scale, and a sensor attached to a lightweight armband to be worn on the nondominant arm tracking basic biometric data (heart rates, oxygenation, and steps taken). As described, CHWs were trained to assist patients with technology setup and troubleshooting. Any unreconciled technical difficulties were addressed by research study staff and the platform vendor as needed. An artificial intelligence algorithm within the mobile app generated a daily score along with alerts sent to the CHW team dashboard, indicating if participants were at or moving away from their clinical baseline in terms of symptoms, biometrics, and functionality. This team dashboard was used in conjunction with a color schematic described (Figure 3). Any scores or alerts indicating that participants were moving away from their baseline were discussed with a CHW project manager. When indicated, CHWs notified clinical team staff during weekday office hours within 2 hours of a biometric or other clinically related concern (ie, significant change in heart rate, blood pressure, body weight, or patient-reported symptoms). Participants were instructed to engage with clinical care teams or urgent or emergent care as they would normally if they experienced symptomatic changes or other concerns outside weekday hours of operation.

Participants were contacted routinely by CHW staff to review medication adherence, nutrition, physical activity, symptoms, and clinic appointments and discuss any unmet social needs as described in the control treatment arm. All elements of other CHW outreach activities were performed as described in the control arm.

### Data Collection and Measures

All study participants completed an enrollment questionnaire focused on health habits and patient experience with home self-care. This questionnaire was developed based on prior patient qualitative interviews and CHW focus groups with those caring for patients with HF [37,38]. These questions were adapted by study investigators for interviewer-assisted administration with inpatients prior to hospital discharge. We used a qualitative process to identify core domains through key informant interviews with patients, community-based primary care physicians, cardiologists, and internal medicine hospitalists. This was coupled with a review of the literature on patient experience in hospitalized settings along with consultations with survey and health services research experts. Draft surveys were pretested with 3 patients with the opportunity for revision prior to study administration. The enrollment questionnaire included 59 items from 7 distinct categories: health-related habits, understanding of the care plan, smartphone knowledge, quality of life (Kansas City Cardiomyopathy Questionnaire) [40], perceptions of physical and mental health, unmet social needs, loneliness (Three-Item Loneliness Scale) [40], and depression (Patient Health Questionnaire-2) [40]. Additional domains included confidence in the ability to perform self-care after discharge and patient-predicted likelihood of readmission within 30 days. Open-ended questions were asked to patients regarding anything that would help them manage their health outside the hospital.

An exit questionnaire administered at the study end (after 30-day enrollment) was also developed. This questionnaire mirrored domains in the enrollment questionnaire to assess any changes (eg, health-related habits and care plan understanding) associated with intervention or control arm assignments. Intervention participants also completed an acceptability questionnaire focused on the digital platform (adapted from components of the Technology Assessment Model Measurement Scales [41] as described by Ben-Zeev et al [42]). Similarly, the exit questionnaire was initially pretested with 3 patients, and no additional changes were made (all pretested questionnaire data will be included in the final analysis).

Separately, participants also completed an exit interview after completion of the 30-day enrollment period. The exit interview was conducted via phone and prompted participants to describe their experience with the digital platform and CHW staff interactions. Specific questions included: *What was it like to work with a CHW for the last month? What was it like using this technology in your home for the last month?* and *Are there things that could have made your time in the study better for you?* Semistructured interviews occurred via phone at times designated by participants and lasted 5-12 minutes. All semistructured interviews were audio-recorded and transcribed verbatim.

### Process Measures, Source, and Timeframe Associated With Primary Outcomes of Feasibility, Acceptability, and Primary Effectiveness

The primary outcomes of feasibility, acceptability, and preliminary effectiveness will be tracked (Table 1). We will report feasibility outcome measures including daily use rates of the biometric sensor (mean hours per day), the digital blood pressure monitor (mean times per day), the weight scale (mean times per day), and completion of the symptom questionnaire (mean times per day). The acceptability outcome measure will be patient responses to the truthfulness of a statement indicating willingness to use the intervention in the future (response options: very true, somewhat true, or not true). Preliminary effectiveness will be measured by tracking 30-day clinical outcomes (hospital readmissions, emergency room visits, and missed primary care and cardiology appointments). Demographic data and survey item responses were captured in REDCap. These items will be summarized, and univariate analysis will be completed for any domains connected to the outcomes. Structured medical record review data extracted from the EMR will also be captured in the REDCap database.

**Table 1:** Outcome measures or covariates for the digitally enabled CHW^a^ intervention.

Outcomes and process measures		Source	Timing
**Feasibility**			
	Use of the digital platform (wearing the biosensor^b^, use of the blood pressure monitor^c^, use of the digital weight scale^c^)	Platform database	At 30 days
	CHW engagement (number of CHW interactions, types of CHW interactions)	CHW interaction log	At 30 days
**Acceptability**			
	Willingness to use the intervention again	Patient exit questionnaire	At 30 days
**Preliminary effectiveness**			
	30-day readmissions	Electronic health record	At 30 days
	Emergency department visits	Electronic health record	At 30 days
	Missed appointments	Electronic health record	At 30 days

^a^CHW: community health worker.

^b^Hours worn per day.

^c^Number of times recorded per day.

### Statistical Analysis

All standards for trial design, analysis, and reporting will be adhered to per the CONSORT (Consolidated Standards on Reporting Trials) checklist [43]. Univariate analysis will include demographic covariates of participants as well as intervention use frequencies, means, and SDs related to feasibility and acceptability outcomes. For the 30-day clinical outcomes of readmission, ED visits, and missed primary care and specialty appointments, we will use the proportion with any readmissions, emergency visits, or missed clinic visits will be compared between the 2 arms using Pearson ^2^ tests. We will also use a logistic regression model to include other potential predictors of the outcome to improve the precision of the estimate. The number of readmissions, ED visits, or missed appointments will be compared using Poisson models.

For exit interviews, we will use a framework analysis to identify main themes along with verbatim transcription for coding and analysis. Interview transcripts will be uploaded into Dedoose (version 8.3.47b.exe; SocioCultural Research Consultants, LLC). An analytic framework will be developed based on the major domains of the patient interview guide. To help facilitate reliability, 2 members of the research team (JC and NS) will serve as coders and familiarize themselves with the raw transcription data. They will then independently identify key themes raised by respondents. An iterative reapplication of this thematic framework will be used to identify all transcript components mapping to these specific themes. Coders will identify associations between themes, user characteristics, and outcomes for all interviews prior to achieving intercoder reliability. Any discrepancies unable to be resolved through discussion by the 2 coders (JC and NS) will be reviewed by a third researcher with expertise in qualitative data. These methods will be completed in concordance with the COREQ (Consolidated Criteria for Reporting Qualitative Research) checklist standards [44]. All trial procedures will also be in concordance with ClinicalTrials.gov regulations (NCT05130008).

### Ethical Considerations

Institutional review board approval (2018P002014) was obtained from the Mass General Brigham Human Research Committee on September 22, 2020. All patients interested in enrollment participated in an informed consent process with the opportunity to ask questions and review all study procedures, data collection, and data analysis processes (primary and secondary) prior to signing an informed consent form. Privacy and protection standards were upheld with secure and password-protected database storage for all study data (which will be presented in deidentified form on publication). All participants were offered US $250 for remuneration for study participation.

## Results

Between September 2022 and June 2023, enrollment was completed (n=50) with participants from inpatient general internal medicine and cardiology study floors at the MGH. The analysis is expected to be completed by January 2023, with results published in the spring of 2024. The study was approved by the IRB on June 4, 2019.

## Discussion

### Anticipated Findings

Here, we describe the protocol of an RCT designed to assess the feasibility, acceptability, and preliminary effectiveness of a novel digitally enabled CHW intervention for patients managing HF at home. The results of this trial are expected to deepen our understanding of experiential barriers patients with HF patients face while living at home and underline the potential value of a home-based CHW-supported digital platform solution. Assessment of the intervention’s ability to augment patient engagement, adherence to care plans, and impact clinical outcomes will be addressed. Given the rising cost of care for patients with HF and heightened morbidity and mortality, the findings of this study may also carry important economic lessons in terms of designing value-based care that can assist in avoiding preventable readmissions for patients with HF who are at high risk for hospitalization.

This study may also add context to our understanding of how digital interventions for patients with HF and other serious illnesses are best implemented. Numerous barriers to adoption have been well documented, and the results generated from this study may aid in the development of a framework, leading to best practices for addressing individual clinical and social barriers that may arise for patients engaging with digital platforms at home. The use of qualitative exit interviews adds to the patient centeredness of the study by highlighting the patient perspective and experience that may generate important solutions for the home management of HF and for unmet social needs. These results may also demonstrate how home-based CHW care can potentially advance patient use and engagement with digital platforms, adherence to clinical care plans, and connections to clinical homes. These study results may be used to strengthen and inform the feasibility and adoption of future digital interventions. Finally, this study also will provide opportunities to clarify our understanding of which types of interactions and resources provided to certain patients with HF by CHWs are most beneficial with regard to clinical outcomes.

### Comparison to Prior Work

Remote monitoring studies have shown some improvements in outcomes for patients with HF. In a large trial with 1571 patients randomized to remote monitoring versus usual care, intervention patients had fewer days lost to unplanned cardiovascular admissions (4.88% vs 6.64%; 95% CI 0.65-1.0; *P*=.046) and decreased all-cause death rate (7.86 vs 11.34; hazard ratio 0.70, 95% CI 0.5-0.96; *P*=.028). However, most studies of remote monitoring have demonstrated mixed results [6]. A large study randomized 1437 participants to a combined health coaching and telemonitoring intervention for 180 days but showed no significant difference in readmission (50% vs 49%; *P*=.74) [45]. Similar outcomes were seen in other large-scale studies [46-48]. Nevertheless, systematic reviews have been suggestive of improved outcomes. In a systematic review analysis of 25 studies inclusive of structured telephonic support and telemonitoring (n=9332), telemonitoring reduced all-cause mortality (n=3740; RR 0.80, 95% CI 0.68-0.94) and HF-related hospitalization (n=2148; RR 0.71, 95% CI 0.6-0.83) [20]. However, all-cause 30-day readmission rates were unchanged. Concordantly, in a meta-analysis of 21 analyzed RCTs assigning patients to different remote patient monitoring interventions (n=6317) with medical personnel support or telephone support, results showed that those interventions that included staff delivering some home-based support (either clinically or administratively) reduced mortality (HR 0.77, 95% CI 0.55-1.08) [49].

While the majority of CHW studies are focused on cancer, there is a growing evidence base demonstrating the impact of CHWs on chronic disease and HF populations. In an RCT (n=426) pairing CHWs with patients who have chronic disease insured by Medicaid at the time of hospital discharge, adherence to posthospital care was improved (60% vs 47%; *P*=.02) [10], but no difference in 30-day readmissions was seen. Another RCT that focused on patients with 2 or more chronic diseases (n=322) demonstrated a reduction in systolic blood pressure associated with a 6-month CHW postdischarge intervention (–11.2 mm Hg vs 1.8 mm Hg; overall *P*=.08) [11]; a 28% reduction in 1-year hospitalizations was also seen, although it did not achieve statistical significance. A large RCT (n=525) testing a nurse practitioner and CHW intervention among patients with cardiovascular disease showed reductions in reduced total cholesterol (difference 19.7 mg/dL), low-density lipoprotein cholesterol (difference 15.9 mg/dL), triglycerides (difference 16.3 mg/dL), systolic blood pressure (difference 0.2 mm Hg), and glycated hemoglobin (difference 0.5%), as compared to enhanced usual care [50]. Some smaller studies have also supported these outcomes. In a study with 28 patients with HF paired with CHWs at the time of hospital discharge, participants paired with a CHW for 12 months saw a decrease in HF-related ED visits (0.71 vs 0.18; *P*<.001), and an 89% decrease in HF readmission (0.64 vs 0.07; *P*<.005) [51]. No significant difference in 30-day readmissions or ED visits was seen compared to matched controls. Systematic review data have also been positive. In a meta-analysis of 16 CHW-focused RCTs, 5 RCTs demonstrated a significant reduction in ED visits (23%-51% reduction; *P*=.05), hospitalizations (21%-50% reduction; *P*<.05), and urgent care visits [52]. We expect that this study will build on this knowledge by filling knowledge gaps related to adoption and adherence to a digitally enabled CHW intervention that has yet to be studied in a clinical trial powered to test its effect.

### Strengths and Limitations

This methodology describes a small pilot trial performed at a single, urban, academic health care center. Since there are limited survey instruments designed to assess user experience with this particular digital platform, we adapted a prior questionnaire with established generalizability to assess this digital platform [37]. While formal patient-reported outcome measure scales were unable to be incorporated for all patient questionnaire covariates due to burdensome instrument length, Patient Health Questionnaire-2, Kansas City Cardiomyopathy Questionnaire, and the Three-Item Loneliness Scale were included as well as a number of items related to health behaviors and satisfaction used in our previous studies [27]. The study strengths include the RCT design focused on a unique intervention for an at-risk cohort of patients facing a burdensome and morbid condition. We also believe that the inclusion of feasibility as an outcome adds strength to the structure and actionability of the trial methodology in 2 ways: by further informing implementation science and strategic deployment of the intervention and by identifying potential barriers and facilitators of this intervention delivery and engagement for future larger trial performance. Additionally, the use of patient response data gathered from questionnaires and exit interviews adds a quasi-mixed methods design element that not only contributes to our understanding of the patient experience with living with HF at home but can also add depth and context to the study outcomes. While this trial will not be adequately powered to assess the true effectiveness of the intervention and our sample size will be limited to a single academic institution that may not be generalizable to other settings, the methodological quality and integrity of the study performance may generate preliminary results that may later be tested in a large multisite clinical trial powered to assess the intervention’s effect on clinical outcomes in larger populations.

### Conclusions and Future Directions

The findings of this trial are expected to contribute to the implementation of digital platforms, as well as the role of CHWs in supporting digital care for a patient with HF. In this way, these findings may improve the performance of a large-scale and multisite RCT and help determine the true effectiveness of this intervention with regard to clinical outcomes. In addition, this trial may answer important questions about what types of CHW interactions can offer the greatest value for patients relevant to demographic, clinical, and social domains. As total medical expenditures for those affected by HF are expected to grow exponentially in the next decade, creating value-based and patient-centered solutions is a fundamental priority for health care institutions and stakeholders alike.

## Abbreviations

CHW: community health worker
CONSORT: Consolidated Standards on Reporting Trials
COREQ: Consolidated Criteria for Reporting Qualitative Research
ED: emergency department
EMR: electronic medical record
HF: heart failure
MGH: Massachusetts General Hospital
RCT: randomized controlled trial
REDCap: Research Electronic Data Capture

## Data Availability

The data sets generated and analyzed during this study are not publicly available due to the small number of participants at a single institution but are available from the corresponding author on reasonable request.
